# Mammary Inflammation

**Published:** 1853-12

**Authors:** Willis Danforth

**Affiliations:** Oswego, Ill.


### *Mammary Inflammation.*

BY WILLIS DANFORTH, M. D, OSWEGO, ILL.

Much has been said of the pathological changes which take place in Mammary Inflammation and Abscess; but, so far as I can learn, little has been done in the way of cure. Without entering upon a discussion of the various theories and treatment of these affections, I propose briefly to state—what is already known to many—that Collodion painted over the entire surface of the breast, as soon as it is found to be painful, hot and tumefied, will at once arrest the inflammation. This simple fact is of incalculable importance to every physician. I might report numerons cases in proof of my position, but one—and it may be considered as the type—will suffice.

Mary Ricker was seized on the 12th of August, the tenth day after her confinement, with chills, pains in both breasts followed, from which she suffered considerably until 4 P. M., when I was sent for. I did not, however, see her till ten o'clock in the evening; I found her breasts much swollen, very hot and extremely painful; pulse 110; tongue coated etc. I painted both breasts freely with collodion, and in twenty minutes she expressed herself much relieved. I left her no medicine. I saw her the next morning and found the breasts still painful; another layer of collodion was applied, and this was the last I did for her. The pain abated, and she was entirely free from fever that day. She was quite well the following day. If the case is seen before pus has formed, I believe this mode of termination of the inflammation may *always* be prevented.—*Amer. Lancet.*

